# Cone beam CT for QA of synthetic CT in MRI only for prostate patients

**DOI:** 10.1002/acm2.12429

**Published:** 2018-09-04

**Authors:** Emilia Palmér, Emilia Persson, Petra Ambolt, Christian Gustafsson, Adalsteinn Gunnlaugsson, Lars E. Olsson

**Affiliations:** ^1^ Department of Hematology, Oncology, and Radiation Physics Skåne University Hospital Lund Sweden; ^2^ Department of Translational Medicine, Medical Radiation Physics Lund University Malmö Sweden

**Keywords:** cone beam CT, MRI, MRI only, prostate cancer, quality assurance, synthetic CT

## Abstract

**Purpose:**

Magnetic resonance imaging (MRI)‐only radiotherapy is performed without computed tomography (CT). A synthetic CT (sCT) is used for treatment planning. The aim of this study was to develop a clinically feasible quality assurance (QA) procedure for sCT using the kV‐cone beam CT (CBCT), in an MRI‐only workflow for prostate cancer patients.

**Material and method:**

Three criteria were addressed; stability in Hounsfield Units (HUs), deviations in HUs between the CT and CBCT, and validation of the QA procedure. For the two first criteria, weekly phantom measurements were performed. For the third criteria, sCT, CT, and CBCT for ten patients were used. Treatment plans were created based on the sCT (MriPlanner^TM^). CT and CBCT images were registered to the sCT. The treatment plan was copied to the CT and CBCT and recalculated. Dose–volume histogram (DVH) metrics were used to evaluate dosimetric differences between the sCT plan and the recalculated CT and CBCT plans. HU distributions in sCT, CT, and CBCT were compared. Well‐defined errors were introduced in the sCT for one patient to evaluate efficacy of the QA procedure.

**Results:**

The kV‐CBCT system was stable in HU over time (standard deviation <40 HU). Variation in HUs between CT and CBCT was <60 HU. The differences between sCT–CT and sCT–CBCT dose distributions were below or equal to 1.0%. The highest mean dose difference for the CT and CBCT dose distribution was 0.6%. No statistically significant difference was found between total mean dose deviations from recalculated CT and CBCT plans, except for femoral head. Comparing HU distributions, the CBCT appeared to be similar to the CT. All introduced errors were identified by the proposed QA procedure, except all tissue compartments assigned as water.

**Conclusion:**

The results in this study shows that CBCT can be used as a clinically feasible QA procedure for MRI‐only radiotherapy of prostate cancer patients.

## 1 INTRODUCTION

The traditional radiotherapy workflow for prostate cancer is built upon two imaging modalities, that is, computed tomography (CT) and magnetic resonance imaging (MRI). The CT data set provides Hounsfield Units (HUs), which the treatment planning system converts to relative electron densities (RED) needed for the absorbed dose calculations. MRI is preferred as a complementary imaging modality due to the superior soft tissue contrast, and therefore enables a more accurate target delineation.^1^ A coregistration of the two data sets is used to create a foundation for target delineation. Although this is a common procedure, uncertainties associated with the MR–CT registration has been estimated to 2 mm for prostate cancer patients.^2^

Interest has been directed toward a workflow using MRI as the only imaging modality for treatment simulation — often denoted as an MRI‐only workflow. An MRI‐only workflow will not only avoid registration prior target delineation, but also simplify the workflow, reduce the workload and therefore become more cost effective.^3^ However, to enable treatment planning from an MRI data set the need for RED required for absorbed dose calculation must be considered.

There are many different methods available for converting MRI data to create a so‐called synthetic CT (sCT).^4^, ^5^, ^6^, ^7^, ^8^, ^9^, ^10^, ^11^, ^12^ An sCT is an image that looks very similar to a CT image and can be used by the treatment planning system for absorbed dose calculations as well as provide digital reconstructed radiographs (DRR). Recently, different methods for generating sCT data set were reviewed.^13^ At present there are two vendors that provide sCT data sets for the male pelvis region (MRCAT^TM^ and MriPlanner^TM^).^11^, ^12^

In a recent multicenter, multivendor study (MR‐only prostate external radiotherapy (MR‐OPERA)), the MriPlanner^TM^ was validated for the male pelvis.^14^ Dose–volume histogram (DVH) comparisons between sCT and CT dose distributions resulted in mean dose deviations that were below 0.3% for all DVH metrics and therefore negligible compared with other uncertainties in radiotherapy. No major errors were found in any of the steps of the sCT image conversion. Although the method is validated, there might be conditions where the sCT data generation is inadequate due to other circumstances, such as operator noncompliance with the MR protocol or unexpected anatomic structures. Therefore, an independent quality assurance (QA) procedure of the sCT data would be preferred before, or in connection with, the first treatment fraction.^13^, ^14^, ^15^ Recently, the cone beam CT (CBCT), which is performed on the treatment machine, was suggested as a way to perform patient specific QA for radiotherapy of the brain.^16^ Assuming that the CBCT data set gives similar attenuation information as a CT, the information can be used to validate the sCT, with respect to HUs. The brain is a rather fixed anatomical geometry, while we expect the male abdomen to be less stable between the planning session and the treatment. Therefore, we need to study if CBCT can be used for QA of sCT data for prostate cancer patients. In addition, it would be of interest to go one step further and verify the sCT data with respect to absorbed dose using CBCT data.

The aim of this study was to develop a clinically feasible QA procedure for MRI‐only radiotherapy of prostate cancer patients using the CBCT to detect errors in a synthetic CT.

## 2 METHODS

In this work, an MRI only workflow using a sCT generated from a standard T2‐weighted MRI generated by MriPlanner^TM^ (Spectronic Medical AB, Sweden) was considered. The workflow is based on the following steps; first an MRI encompassing the outer body contour, large field of view (FOV) MRI, is acquired along with sequences dedicated for delineation and fiducial marker identification. A sCT is generated based on the large FOV MRI through an automatic generation process directly connected to the MR scanner and treatment planning system. The sCT is used in the same manner as a conventional CT throughout the treatment planning process. The MRI is used for delineation of target and organs at risk (OAR) and fiducial marker identification, without the need for image registration toward the sCT. A treatment plan is created and dose is calculated using the sCT. The patients are scheduled for the first treatment fraction approximately 2 weeks after the MRI. In our suggested workflow a CBCT is acquired at the first treatment fraction and the QA procedure can be performed before the second treatment fraction. The patients are positioned using gold fiducials markers and the CBCT is solely to be used for QA purposes.

In order to use the CBCT data as a QA tool for sCT data the following criteria need to be addressed:

1. Stability in HU over time for the CBCT system used. To establish that the CBCT system does not drift over time, introducing systematic errors if not corrected for when dose calculating.
2. Deviation in HU between CT and the CBCT system used. To investigate the need of using specific HU to RED calibration curves.
3. Validation of the QA procedure using a relevant patient data set. To investigate the feasibility for dose recalculation on CBCT data.

### 2.A Phantom measurements

Addressing criteria 1), periodic measurements were carried out to evaluate the stability of the system used for kV‐CBCT imaging. The kV‐CBCT system used was Varians On‐Board Imager^TM^ TrueBeam^TM^. Measurements were performed weekly on one treatment unit for 8 weeks using the CIRS Model 062M electron density phantom (Computerized Imaging Research Systems Inc., Norfolk, VA, USA). The mean HU of the phantom inserts were evaluated in the registration module Eclipse Image Registration (Varian Medical Systems v. 13.6, Palo Alto, CA, USA). Addressing criteria 2), one phantom measurement was performed on the CT scanner Siemens Somatom Definition AS+. The mean HU of the phantom inserts were compared between the CT and CBCT units used in this study.

### 2.B Patients

The patients in this paper were from an ongoing study (MR‐only prostate radiotherapy excluding CT (MR‐PROTECT)), which studies a radiotherapy workflow incorporating all mentioned advantages of using an MRI‐only routine. The study has been approved by the Swedish ethics review board in Lund (Dnr 2016/1033). Between April and September 2017, ten consecutive patients were included and received an MRI‐only treatment. The patients were prescribed 78 Gy over 39 fractions to the prostate, with daily patient positioning based on gold fiducials with orthogonal kV images. The initial ten patients were the study population used in this paper. They had a median (range) age of 72.5 (61–81) years and median (range) weight and height of 86.5 (62–95) kg and 174.5 (165–195) cm.

### 2.C Imaging

**Figure 1:**
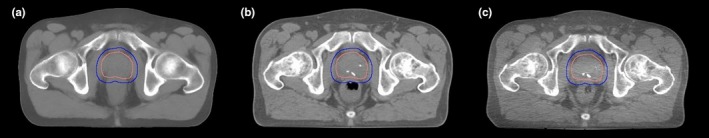
Illustration of one transversal slice in the three data sets for the same patient. From the left sCT (a), CT (b), and CBCT (c) data sets. The outer delineation (blue) represents the planning target volume (PTV) and the inner delineation (pink) the clinical target volume (CTV). The white dots seen in the CT and CBCT slices are calcification in the prostate.

The patients underwent MR and CT imaging prior to their treatment, and a CBCT image was acquired at the first treatment fraction. CBCT was solely acquired for QA purposes and the patients were positioned daily using gold fiducial markers during the whole course of treatment including the first fraction. The conversion software MriPlanner^TM^ was used for sCT generation.^12^ In the MR‐PROTECT study, the CT data set was primarily acquired to enable checks and validation of the MRI‐only workflow. The primary use of the CT data in this presenting study was to validate the dose calculation ability in CBCT data. Images of sCT, CT, and CBCT are given for one patient (Fig. 1).

The MR scanner and field strength used was GE Discovery, 750 w 3.0 T with a flat table top. Patient fixation included knee, ankle support, and reference tattoos to enable a reproducible patient positioning. The receiver coil used was a 16‐channel GEM anterior array placed on a stiff coil bridge, ensuring no deformation of the outer patient body contour. The sequence used for the sCT generation has previously been described.^14^, ^17^ The sCT conversion software required a T2‐weighted MRI data set with a FOV enclosing the complete patient contour. The cranio‐caudal extent of the MRI was set to include the target area (i.e., the prostate) and the OARs, including rectum and bladder.

The CT was acquired directly after the MRI using equivalent patient fixation, using the same CT scanner used for phantom measurement. The CBCT data set was acquired at the first treatment fraction, approximately 2 weeks after CT and MR imaging, using the same kV‐CBCT imaging device as for the phantom measurements. Equivalent patient fixation was used as for both MR and CT imaging. The reference tattoos made at the MR were used to reproduce the patient position during both CT and CBCT imaging. Parameter settings for MR, CT, and CBCT imaging are given in Table 1.

**Table 1:** The acquisition parameters for MR, CT, and CBCT data sets

Acquisition parameter	MR scan	CT scan	CBCT scan
Slice thickness (mm)	2.5	3.0	2.0
kV		120	125
Fan type			Half
Trajectory			Full
Sequence type	Fast recovery FSE		
2D/3D	2D		
Freq. FOV right–left direction (mm)	448		
Phase FOV anterior–posterior direction (mm)	314		
Scan matrix (freq. × phase)	640 × 512		
Recon. matrix (freq. × phase)	1024 × 1024		
TR (ms)	15 000		
TE (ms)	96		
Slice spacing (mm)	0		
Number of slices	88		
Number of echoes	1		
3D geometry correction	On		
Bandwidth (Hz/Pixel)	390		
Shimming method	Auto (first order)		
RF transmit mode	Multi transmit		
Acquisition time (min)	7		

### 2.D Treatment plan recalculation

The original treatment plans in this study were created based on the sCT data. Target and OARs were contoured manually using the MRI. CT and CBCT data were automatically matched rigidly, excluding rotation, based on bony anatomy toward the sCT in Eclipse. Target volumes and OARs were transferred from the sCT data set to the CT and CBCT data set. The body contour structure was separately generated for each data set. According to the clinical protocol, any connected areas of air in the rectum exceeding 4 cm in diameter in any direction is not acceptable for treatment planning. Therefore, if such areas were present in the CT and/or CBCT data set, these were replaced by water. The sCT had, per definition, no air inserted in the conversion of the rectum. The clinical sCT plan was copied onto the CT and CBCT data set and recalculated. As a result there were three treatment plans, based on three different images, with identical beam setup and identical monitor units. The HU to RED conversion used clinically at Skåne University Hospital was applied to all data sets to enable absorbed dose calculations.

DVH metrics used for definition of dose constraints were PTV D_mean_, PTV D_98%_, PTV D_95%_, CTV min, rectum D_30%_, rectum D_15%_, rectum D_10%_, bladder D_mean_, left femoral head D_2%_, right femoral head D_2%_, and body D_0.1%_. These were used to evaluate dosimetric differences between the original sCT plan and the recalculated CT and CBCT plans.

A statistical analysis was carried out to investigate if there was a significant difference between the total mean dose deviations for the DVH metrics, resulting from recalculations on CT and CBCT data. An independent two‐tail, two‐sample t‐test, assuming equal variance, with a 5% significance level was performed using MATLAB R2017a (MathWorks, Inc., Natick, MA, USA) and the resulting confidential intervals (CI) were evaluated.

In two patients, representing the best and worst case with respect to CT/CBCT PTV D_mean_ difference, comparison of the HU distributions in sCT, CT, and CBCT were carried out. To enable a fair comparison of HU distributions, all data sets for these patients were recalculated to have the same resolution and slice thickness.

### 2.E Simulation of sCT data with errors

**Figure 2:**
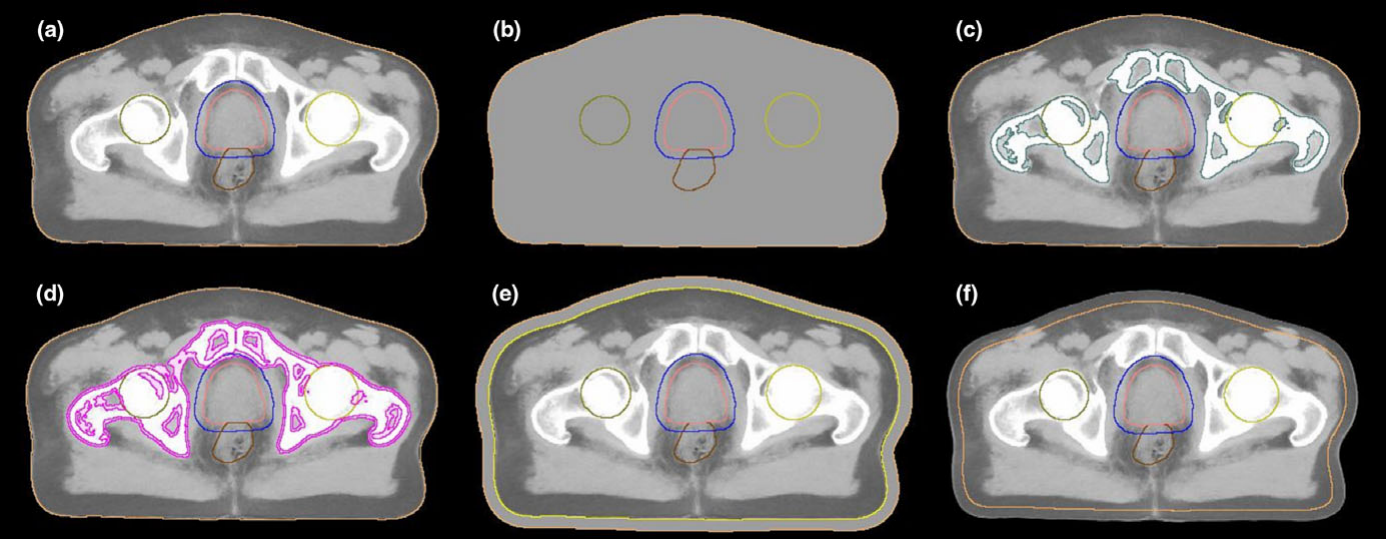
Illustration of the original sCT and the sCTs with errors introduced. (a) original sCT, (b) all tissue assigned as water, (c) bone structure assigned as cortical bone, (d) enlarged bone structure with 2 mm cortical bone, (e) enlarged patient body contour with +10 mm water and (f) decreased patient body contour with −10 mm. The delineations in images are PTV (blue), CTV (pink), femoral head (yellow) and rectum (brown).

In order to assess the capability of the method to detect sCT/CBCT mismatch, errors were intentionally introduced in the sCT data set. This was performed on one patient having the smallest dose difference in PTV D_mean_ between the original sCT plan and the recalculated CBCT plan (−0.03%). The following errors were introduced: all tissue compartments assigned as water, the complete bone structure assigned as cortical bone, uniformly (2 mm) enlarged bone structures, and changes in patient body contour dimension of ±10 mm (Fig 2). The extended bone structure was assigned cortical bone and extended patient body contour to water. For each new sCT data set impaired with an error, a treatment plan was created and optimized according to local clinical protocol. The new calculated plan was transferred onto the original sCT and CBCT data sets and absorbed dose was recalculated. This resulted in three treatment plans based on three different images for each introduced error. PTV D_mean_ was used to compare dose differences between the plan based on sCT with error and the recalculated sCT and CBCT plans.

## 3 RESULTS

Phantom measurements showed that the kV‐CBCT system was stable in HU over time. The standard deviation (SD) ranged from 5.9 to 40 HU for materials with 0.2–1.7 RED. There was less than 60 HU variation between CBCT and CT data.

**Figure 3:**
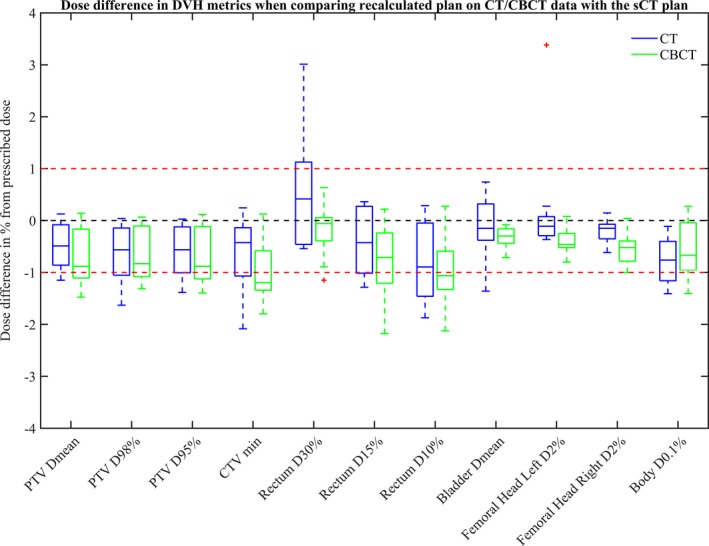
Deviations in percentage between dose distributions calculated on sCT and CT data sets (CT–sCT, blue) and between sCT and CBCT data sets (CBCT–sCT, green). The DVH metrics evaluated were for PTV, CTV, rectum, bladder, femoral heads, and body.

For all patients, a successful dose recalculation was performed based on the CBCT data sets using the standard clinical HU to RED conversion. Recalculations based on CT and CBCT data showed similar deviations from the sCT data calculation. The results are described as dose differences in percent of prescribed dose for the clinical protocol DVH metrics, comparing CT and CBCT dose distribution to the sCT dose distribution for ten patients (Fig 3). The baseline of zero represents no difference between the DVH metrics compared.

There were only small deviations for all DVH metrics between the sCT and CT dose distributions. Median doses were within 0.9% deviation for all metrics. The deviations ranged from −2.1% to 3.4%. An outlier was found for the DVH metric left femoral head D_2%_ and had a value of 3.4%. Rectum D_30%_ was the DVH metric that showed the largest overall deviation with a range of −0.5% to 3.0%.

Generally, there was a larger median deviation in the investigated DVH metrics for the CBCT versus sCT dose distribution compared to CBCT versus CT. Two median doses, CTV min and rectum D_10%_, were below the ‐1% deviation. The deviations ranged from −2.2% to 0.6%, a slightly smaller range than seen for the CT comparison. An outlier for the CBCT data set could be seen for the DVH metric rectum D_30%_ with a value of ‐1.1%. The outlier seen for the femoral head in the CT comparison was not present in the CBCT comparison.

Table 2 shows the total mean absorbed dose difference in percent of prescribed dose for the clinical protocol DVH metrics for ten patients and the corresponding SD. Mean dose differences between sCT and CT dose distributions were below or equal to 0.8% for all DVH metrics. The respective mean dose differences between sCT and CBCT dose distributions were below or equal to 1.0%. There was a significant difference between CT and CBCT in the right femoral head D_2%_. All other investigated DVH metrics showed no significant difference between recalculated CT and CBCT plans.

**Table 2:** Mean dose deviation between sCT and CT and between sCT and CBCT for ten patients

Prescription	CT – sCT (% of prescribed dose)	SD	CBCT – sCT (% of prescribed dose)	SD	CI
PTV D_mean_	−0.5	0.5	−0.8	0.6	[−0.2 0.8]
PTV D_98%_	−0.6	0.5	−0.7	0.5	[−0.4 0.6]
PTV D_95%_	−0.6	0.5	−0.7	0.5	[−0.4 0.6]
CTV min	−0.6	0.7	−1.0	0.6	[−0.3 1.0]
Rectum D_30%_	0.5	1.1	−0.1	0.5	[−0.1 1.5]
Rectum D_15%_	−0.4	0.6	−0.8	0.7	[−0.3 1.0]
Rectum D_10%_	−0.8	0.8	−0.9	0.7	[−0.6 0.8]
Bladder D_mean_	−0.1	0.6	−0.3	0.2	[−0.2 0.7]
Left femoral head D_2%_	0.2	1.1	−0.4	0.2	[−0.1 1.4]
Right femoral head D_2%_	−0.2	0.2	−0.5	0.3	[0.0 0.6]
BODY D_0.1%_	−0.8	0.5	−0.6	0.6	[−0.6 0.4]

**Figure 4:**
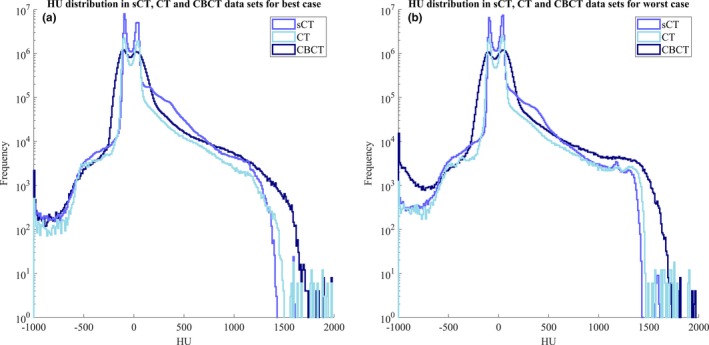
HU distribution for sCT (blue), CT (light blue), and CBCT (dark blue) data sets acquired for two patients, best case (a) and worse case (b).

Figure 4 shows HU distributions in sCT, CT, and CBCT data sets for two patients. These represented the best (left) and worst case (right) in terms of differences in PTV D_mean_ deviation between CT and CBCT dose calculations. The best case had a difference of 0.01% for the PTV D_mean_ and the worst case a 0.6% difference.

In general, the HU histograms overlap for all data sets. A local overlap could clearly be seen in the muscle and fat region, representing the peaks, in Fig. 4. The CBCT distribution seemed to have a more level appearance compared to the other distributions.

Table 3 presents the results comparing PTV D_mean_ between plans from sCT with errors and plans recalculated for sCT and CBCT. The differences are presented in percentage of the prescribed dose. The deviations ranged from −3.4% to 3.7%. The largest dose difference was found for the sCT with an enlarged patient body contour in both comparisons. The recalculated CBCT plan had a similar dose difference for all evaluated errors compared to the recalculated sCT plan.

**Table 3:** PTV D_mean_ difference between error sCT and recalculated sCT and CBCT plans for one patient

Error introduced	sCT – sCT with error (% of prescribed dose)	CBCT – sCT with error (% of prescribed dose)
sCT water	−0.9	−0.8
sCT cortical bone	−3.4	−3.4
sCT enlarged bone	1.8	1.9
sCT enlarged body	3.6	3.7
sCT decreased body	−3.2	−3.1

## 4 DISCUSSION

The present study investigates the prerequisites for using CBCT as a clinical QA tool for the sCT in an MRI‐only workflow of prostate radiotherapy. The suggested QA procedure includes dose calculations on CBCT data sets with a standard HU to RED conversion. Dose recalculation on CBCT data sets would enable a verification of the generated sCT and detection of errors. CBCT imaging is a routine technique used on treatment machines for patient positioning and would enable a clinically practical QA tool in MRI‐only workflows.

Our method relies on the consistency in HU of the specific CBCT system. The CBCT system used in this study was shown to be stable over time. If the CBCT HU would drift over time, a possible error could be introduced in the QA if not corrected for in the dose calculation. In addition, our CBCT system was shown to be equivalent to the CT in terms of HU, and therefore no corrections for the HU to RED were needed. This made our method of dose calculations on CBCT data straight forward. It has been stated that, depending on the manufacturer of the CBCT imaging system, various results for the CT equivalence of the CBCT HU are to be expected.^18^ One should be aware of that different CBCT systems may need correction prior to the dose calculations. The use of different HU to RED corrections has previously been considered a confounding factor in MRI‐only treatment planning.^19^

Recalculation of the clinical sCT treatment plans on both CT and CBCT data sets resulted in small variations in dose differences, with a median within 1% for both data sets. The range of differences for the dose comparisons differed between CT and CBCT dose recalculations. The differences were mainly due to an outlier in the left femoral head D_2%_ and a higher deviation in rectum D_30%_ for the CT comparison. Other DVH metrics for the dose comparisons had more comparable ranges of dose differences. The mean dose differences seen for CT and CBCT dose recalculations were similar with the highest difference of 0.6% also seen for the left femoral head D_2%_ and rectum D_30%_. No significant difference was found for 10 out of 11 DVH metrics evaluated. Significant difference was found in the right femoral head D_2%_. This could be due to difference in femoral head positioning between CT and CBCT imaging. Regardless, dose deviation between the comparisons for this metric was small and would have resulted in the same clinical decision.

The outlier seen for the left femoral head D_2%_ was caused by a small cavity in the patients left femoral head with a large HU difference compared to surrounding cancellous bone tissue. This cavity was not present in the generated synthetic CT. This resulted in a dose deviation in this DVH metric. No clinical impact on the overall DVH appearance and PTV D_mean_ was found, but was reflected in the point dose of the femoral head. In the CBCT data set, the cavity was depicted in the images but was not present in the dose comparison. We hypothesize that this is due to the higher scattering in the CBCT data set, resulting in a smoothing effect of the cavity in the CBCT data set. The rectum DVH metrics had a larger overall range of dose deviations, which is a phenomenon that has been observed previously.^14^ This could be explained by the presence of air cavities in the rectum, which are not generated in the synthetic CT, for the reasons outlined above.

In a previous publication validating dosimetric accuracy and clinical robustness of MriPlanner^TM^, an overshoot was found between CT and sCT comparisons,^14^ which was assumed to be an effect of body relaxation due to the longer examination time of MRI compared to CT. In contrast to the MR‐OPERA study, where the original treatment plan was based on CT, our study has a clinical treatment plan based on the sCT. This should result in the same behavior in the opposite direction, which was demonstrated in Fig. 3 in the dose comparison of both CT and CBCT, excluding the CT rectum D_30%_ comparison. Although efforts have been made to reduce the length of the MR protocol in the present study, the MR examination times is inevitably longer, and anatomical changes can still be present.

Dose calculations on CBCT data sets have been investigated earlier, but the aim of these studies is often directed toward clinical dose calculations. In contrast to these studies investigating adaptive planning, our study aims at using the CBCT based dose calculation as a QA tool in an MRI‐only workflow. To use CBCT data set for clinically adaptive planning has very different demands compared to using it as part of a QA program. Previous studies investigating the dosimetric feasibility of CBCT‐based treatment planning showed that a HU to RED correction is necessary to obtain an accurate dose distribution.^20^, ^21^ Others state that an accurate dose distribution could be obtained using the standard HU to RED conversion curve. Yoo et al.^22^ compared CBCT and CT based IMRT treatment plans relevant to adaptive radiation therapy of prostate patients. The dose distributions on CBCT and CT were in good agreement and the deviation seen was due to setup error. de Smet et al.^18^ compared dose calculations on CBCT and CT data sets with the aim of using the CBCT for treatment evaluation in case of anatomical changes and adaptive planning. The result for the Varian On‐Board Imager™ TrueBeam™ CBCT system using the standard HU to RED conversion curve was a 2%–3% difference in dose distribution.

The use of CBCT imaging as a QA tool would not necessarily have to result in dose calculations that are clinically acceptable for treatment delivery, as needed for adaptive planning. The method needs to be sensitive enough to detect errors in the sCT data. To incorporate the advantages of MRI‐only, a need for a CT independent QA are desired. Previously, a method to verify the sCT data using the CBCT data was presented.^16^ By creating HU to RED conversion curves based on CBCT data sets and statistically analyzing median values of the binned absolute errors (MeAE), it was concluded that verification of the sCT data was possible with CBCT data using a population based calibration curve. In contrast to the previous study, we chose to make dose calculations based on the CBCT data without corrections to the HU to RED curve. The reason behind that decision was the need for finding a clinically feasible QA tool that could be easily incorporated in the clinical workflow. Without correction of the HU to RED and with a simple recalculation, we have found that the CBCT based dose recalculation is equivalent to recalculation performed on CT data.

As an attempt to test the QA procedure, we chose to introduce simple errors in our sCT data. The result from this simulation showed that our suggested method could detect our introduced errors except when all tissue was assigned as water. The recalculation on the original sCT and CBCT data resulted in similar results indicating that the CBCT QA tool could accurately detect the introduced error. Setting all tissues to water does not introduce large dosimetric errors in the pelvis area, which is in accordance with our result.^23^ The results in our study indicate that fairly large errors must be introduced in order to reach absorbed dose deviations above 1%. The simulation of sCT data with errors performed in this study aimed at demonstrating the feasibility of using CBCT data as a QA tool in an MRI‐only workflow. In order to make a full evaluation of the sensitivity of the QA tool, a more thorough simulation is needed, and recommendations on action levels developed. The action levels will depend on the impact the error will have on the delivered dose to the patient and would probably be site‐specific. Careful evaluation of the potential errors that could be introduced in a sCT and their significance must be considered, although this was outside the scope of this study.

The MRI, CT, and CBCT data used in this study were acquired at different occasions. The MRI and CT data were usually acquired in close connection (on the same day typically within 60 min), while MRI and CBCT data were acquired within 2 weeks interval. During this time a change in body contour and anatomy are likely to occur. Difference in bladder and rectum filling are likely to affect the result of the dose calculation. In our case, the same structure outlines were used in order to have comparable DVHs for all dose matrixes. Another strategy could have been to recontour target and OARs on the CBCT and the CT. This was avoided in order to not introduce uncertainties associated with recontouring on images with poor soft‐tissue contrast. Repositioning of the patients is a factor that, despite efforts to make a reproducible positioning, can still affect the dose calculations. The impact repositioning has in the evaluation could be minimized by using deformable image registration. Although deformable registration would minimize the impact of repositioning, and probably result in smaller dose deviations, we wanted to develop a method that was currently straightforward and clinically feasible for QA. The use of deformable image registration would mean additional workload for the clinic, since additional software may be needed and the risk of introducing additional uncertainties related to the registration.

The suggested workflow acquires a CBCT at the first fraction of treatment and the QA could be performed prior to the next treatment fraction. To detect gross errors in the sCT this should be sufficient, since very large errors must be present in the sCT to reach a high dose‐deviation, as shown in our error simulations. In case of higher fraction doses, such as hypofractionation, or if a more HU‐sensitive treatment such as protons are used, an alterative workflow could be preferred. In that case, a suggestion could be to schedule the patient to a CBCT imaging directly prior to or after the MRI. This can eliminate potential errors introduced by anatomy changes that could be present if a 2‐week interval between the images is used. Another potential limitation of using CBCT as a QA tool could be the inferior image quality of the CBCT that sometimes introduces artifacts in the images. Artifacts were not seen for any of the patients included in this evaluation. If artefacts are present, one should consider to what extent they could influence the dose calculation. One potential solution to reduce the impact of artefacts is to override the HU in these areas.

The results from our study show, despite no correction of positioning, comparable dose distributions within a few percentage points between recalculated CT and CBCT treatment plans. From the dose calculations based on both CT and CBCT data, comparable assumptions could be made, and both comparisons are within earlier stated criterion for reliable use of MRI only of 2%.^3^ Focusing on dosimetric accuracy, it could be seen that the dosimetric deviation between CBCT and sCT data was smaller than the estimated level of overall dose uncertainty (5%–10%) for external beam radiotherapy.^24^

The proposed QA procedure would benefit from further investigations of the type and magnitude of errors that can be detected. This would require measurements on a larger population and a wider span of introduced errors in the sCT. Future work could include development of a QA procedure for an MRI‐only workflow that does not involve dose recalculations. Although our results show that this is feasible, and in our clinic very straight forward to implement, a more general method applicable to any CBCT vendor and clinic would be beneficial. This could include the comparison of histograms and correlations to recalculated doses such as those produced in this study. With such a database, incoming sCT and CBCT data sets could be compared within the database and a theoretical expected dose deviation achieved.

Prior to the introduction of CBCT as a QA tool of MRI only in the clinic, attention should be paid to the need for using specific calibration curves and consistency in HU of the CBCT system. Therefore, an investigation of stability over time and deviation in HU between CT and CBCT system before applying any QA method based on CBCT data is needed. If several different systems are used for CBCT imaging, the variation between systems and time dependence should also be studied prior to any use of suggested method. Using patients with corresponding CT examinations for validation of the CBCT QA is also recommended. This procedure needs to be repeated for different anatomical regions.

## 5 CONCLUSIONS

The CBCT and CT systems fulfilled the criteria enabling CBCT data to be used as a QA tool for sCT. Dose calculations based on sCT and CBCT data sets were found to have an absorbed dose difference within clinically acceptable criteria. This shows that dose plan recalculation on CBCT data set can be used as a feasible QA procedure for detection of gross errors in MRI‐only radiotherapy of prostate cancer patients. The suggested method has been demonstrated to successfully detect introduced errors in a sCT.

## CONFLICT OF INTEREST

No conflicts of interest.
